# Random forest forecasting of time to failure for Granite hydraulic fracturing using acoustic emission signals

**DOI:** 10.1007/s44421-025-00005-2

**Published:** 2025-10-03

**Authors:** Madeleine P. Hooper, Arnold Yuxuan Xie, Bing Q. Li

**Affiliations:** Department of Civil and Environmental Engineering, Western University, London, Canada

**Keywords:** Geothermal, Hydraulic fracture, Seismicity, Time to failure, Machine Learning

## Abstract

Geothermal energy is a key resource to support carbon–neutral targets for its high energy baseload production. However, its utilization often involves hydraulic fracturing that can induce earthquakes. Accurate forecasting of the timing of these fractures and associated seismicity can inform hazard mitigation strategies where traditional methods often fall in short. Here, we utilize acoustic emission (AE) signals obtained from a series of hydraulic fracturing experiments on Barre Granite under a variety of stress regime to develop a random forest model to forecast the time to failure. The failure time is defined by a large pressure drop denoting the propagation of unstable macro-scale hydraulic fractures to the edges of the specimen. We achieve a coefficient of determination *R*^2^ of 0.97 on test data using 50% of the data as the training set. We find that the first 20 statistical features constitute 100% of the contribution to the forecast, where the top 4 features are mean, minimum and kurtosis of the first finite difference and skewness of the signal voltage. At our given injection rate, our model can accurately forecast 1000 to 1800 s before failure compared to the 18 to 69 s in advance when using a benchmark inverse AE rate model. Our results suggest that it is possible to forecast failure of a rock specimen prior to the onset of accelerated seismic release, with implications for managing induced seismicity hazards.

## Introduction

An increase in the supply of high base-level energy is needed to simultaneously decarbonize and meet increasing energy demands. Geothermal is a suitable candidate to serve such needs because it can provide higher base-load energy levels than other forms of renewable energy [1, 2]. While critical to decarbonization, enhanced geothermal energy systems can cause anthropogenic earthquakes. For example, cold water injection into an enhanced geothermal energy system in Pohang caused a Mw 5.4 earthquake that injured 90 people and caused $75.8 million USD in damages [3, 4]. Therefore, it is critical for operators to minimize the likelihood of large, induced earthquakes, or, failing that, provide sufficient warning of such events to nearby communities.

Traditionally, seismologists use recurrence intervals of past, characteristic earthquakes to forecast that a similar earthquake will occur at the same interval in the future [5]. While commonly used, the recurrence interval method has failed numerous times [5]. For example, researchers analyzed site stratigraphy in Parkfield, California and forecasted a 21.9-year recurrence interval for earthquakes of a certain magnitude with a 3.1-year error bound,however, the method failed when it forecasted the earthquake to occur by 1993 and no similar earthquake occurred until 2004 [6]. Likewise, when modeled on laboratory data, the recurrence interval method only achieves coefficients of determination *R*^2^ up to 0.49 (where 0.0 indicates no linear correlation and 1.0 indicates perfect correlation) [7].

However, recent increases in algorithmic knowledge, computational power, and dataset size have promoted an increased interest in the use of machine learning models for temporal earthquake forecasting [8]. For instance, machine learning models suggest that the fault gouge’s mineral grains emit acoustic emission (AE) signals when the gouge’s neighboring fault blocks shift [9]. As failure approaches, the amplitude of the signals increases, with the greatest change occurring at fault rupture [9].

Rouet-Leduc et al. [9] recorded AE signals emitted during double-direct shear experiments and demonstrated that a random forest model can use the signals to forecast the time to failure of a laboratory fault with a *R*^2^ of 0.89. Building on Rouet-Leduc et al.’s success, Laurenti et al. [10] demonstrated that a deep neural network model can also use AE signals to forecast the time to failure of a laboratory fault with a *R*^2^ of 0.97. Similarly, Wang et al. [11] demonstrated that transfer learning can also use AE signals to forecast the time to failure of a laboratory fault with a Mean Absolute Percentage Error of 2.98%, where the percentage indicates the difference between forecasted and true time to failures. However, in each of these instances, the rock specimen was loaded in a double-direct shear device, and multiple stick–slip events were recorded during a single experiment. Thus, the slip occurs largely as a collection of mode II cracks along the pre-defined plane of weakness [12]. On the other hand, researchers have linked large earthquakes to new fractures created by hydraulic fracturing alongside associated reactivation of existing natural fractures [13]. These hydraulic fractures tend to propagate as a series of mixed-mode I/II cracks [14], and are critical to understand and forecast the associated seismic hazards [15].

Hydraulic fracturing is widely used in the mining and oil and gas industries for pre-conditioning in cave mining, stress relief, and permeability enhancement [16–18]. It is essential for natural gas extraction in low permeability strata, particularly shale [19]. Among the approximately 2 million wells recently drilled in the US, 95% of them involve hydraulic fracturing [20]. As a result, it is important that operators can forecast the time to a large event when hydraulic fracturing is used to stimulate a rock mass [13].

To our knowledge, previous researchers have only applied machine-learning-based time to failure forecasting to stick–slip experiments on pre-existing faults, but not to the hydraulic fracturing of intact rock given the more stringent requirement for numerous experiments to generate a suite of training and testing data. Thus, a research gap exists in if and how machine learning can be used to temporally forecast rock fractures caused by hydraulic fracturing using AE signals.

Here, we employ a similar random forest approach to forecast the propagation of unstable macro-scale hydraulic fractures, which is chosen for its robustness against outliers, physical interpretability, and ease of training. Specifically, we take as inputs AE data collected from a series of fourteen hydraulic fracturing experiments in Barre Granite. The target in each experiment is to forecast the failure time that is defined by a large pressure drop denoting the propagation of unstable macro-scale hydraulic fractures to the edges of the specimen. We compare our model to established methods of forecasting time to failure, such as the inverse AE rate method.

We describe the problem statement and solution methodology in Sect. 2, the experimental procedure in Sect. 3, the results in Sect. 4, and the conclusions and recommendations for future work in Sect. 5.

## Methodology

To validate if machine learning can be used for temporal forecasting of the propagation of unstable specimen-scale rock fractures caused by hydraulic fracturing [21], a set of statistical features are extracted from AE signals acquired during a series of hydraulic fracturing experiments [9, 22]. The statistical features are then used to train a random forest machine learning model to forecast the failure time corresponding to the development of unstable specimen-scale hydraulic fractures [23].

### Experimental setup

The training and testing data used in this research were acquired from fourteen hydraulic fracturing experiments on Barre Granite containing internal flaws with various geometries [22].

**Fig. 1:**
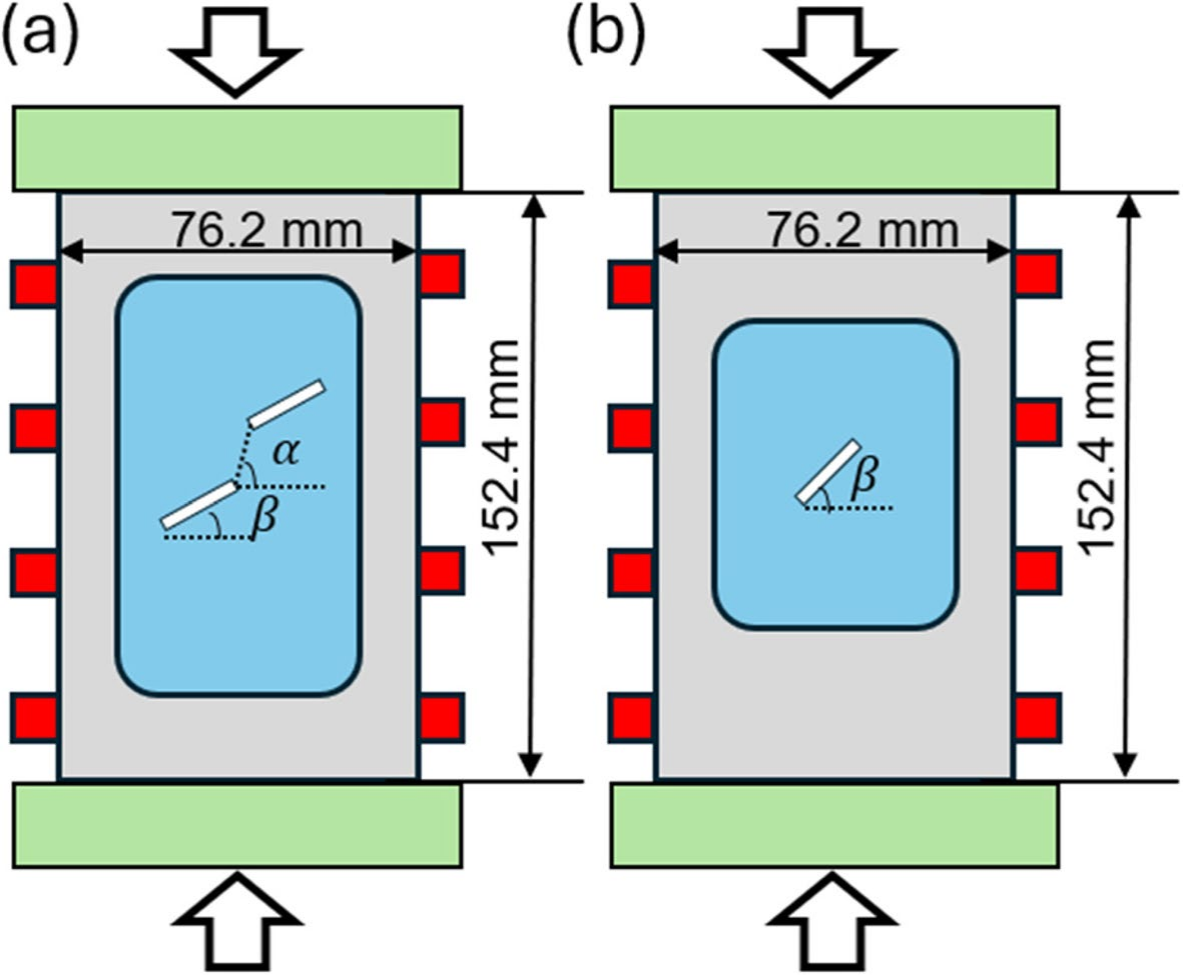
Specimen dimension and sensor layout for (**a**) double-flaw and (**b**) single-flaw experiments. Green blocks are loading platens. Red blocks stand for sensors. Blue area indicates region subjected to water pressure. Flaw length is 1.27 mm. $\beta$ is the flaw inclination angle and *α* is the bridging angle

The experimental setup and dimensions of the specimens are depicted in Fig. 1. The specimens were prepared using a waterjet to cut the internal flaws and the final specimen dimensions of 1″ × 3″ × 6″ (25.4 mm × 76.2 mm × 152.4 mm). Each specimen was subjected to pressurized water using a pressure volume actuator to create hydraulic fractures. The injection pressure was increased in 0.5 MPa increments. A Baldwin load frame pre-loaded the specimens with a vertical confining pressure of 0 MPa or 5 MPa.

Experiments with two flaws are labeled as, for example, “2a-30–120-VL5-INC5-B”, where the flaw spacing is 2a = 1.27 mm, bridging angle $\alpha =120^\circ$ and both flaws are inclined at *β* = 30°. VL5 indicates a vertical pressure of 5 MPa, and INC5 implies the hydraulic injection pressure was increased in 5 MPa steps. The letter ‘B’ at the end is used to distinguish different runs under the same condition. Single-flaw experiments are labelled as, for example, 30-VL5-INC5-B’, where flaw inclination angle *β* = 30° and the remaining naming terms follow the same notation as double-flaw specimens.

For AE signal recording, each pre-cut Barre Granite sample was instrumented with 8 Physical Acoustic Corporation Micro30 s sensors (response spectrum shown in Fig. 1) attached using 0.002 inch double-sided acrylic tape and hot glue [22]. The AE signals are pre-amplified by 20 dB corresponding to a factor of 10 × in voltage and recorded by the PCI-2 data acquisition cards with a sampling rate of 5 MHz continuously to ensure that AE signals were noted both before and during fracturing [22].

### Machine learning

**Fig. 2:**
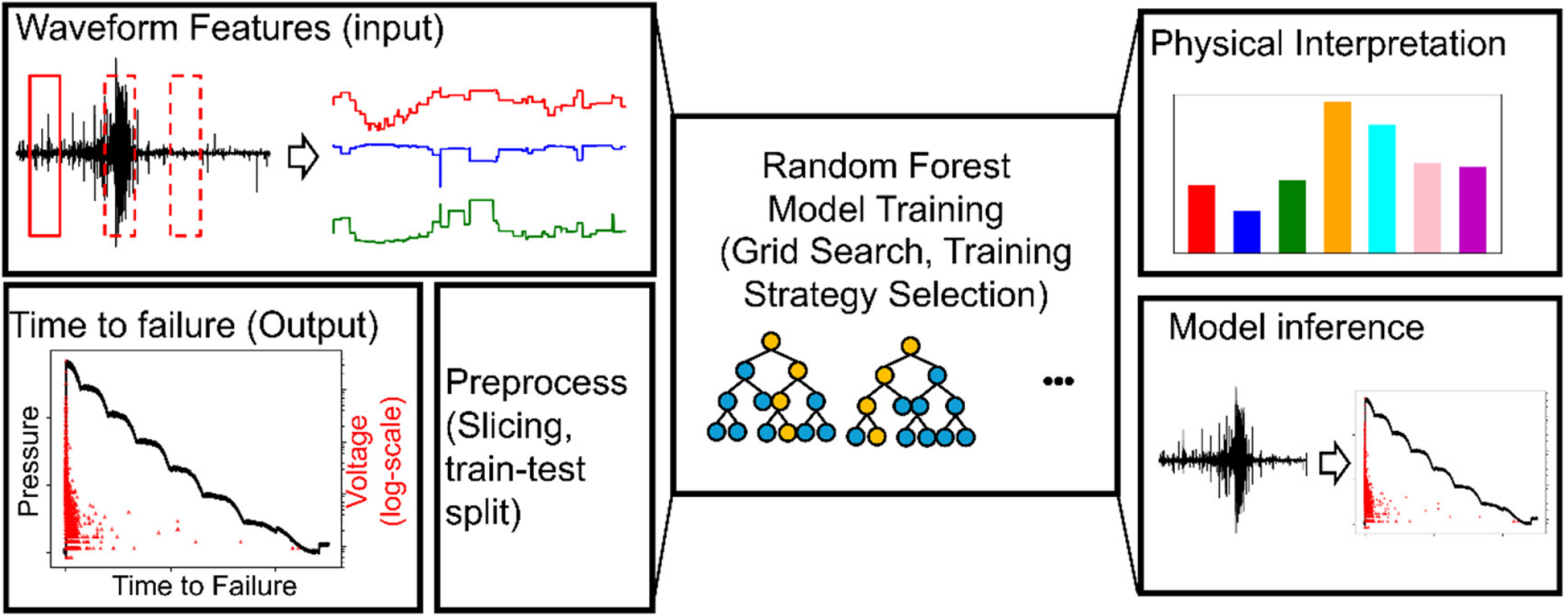
Schematic plot of the workflow

The workflow is summarized in Fig. 2. We choose a random forest model as our major machine learning algorithm for its interpretability. The inputs are statistical features extracted from the raw AE signals, while the output is the time to failure. The inputs and outputs are passed to a series of preprocessing steps for numerical stability of the machine learning model. The trained model can not only serve as a time to failure forecasting model using the raw AE signal data but also provide statistical insight into the physicality of the forecasting.

#### Data preprocessing

**Fig. 3:**
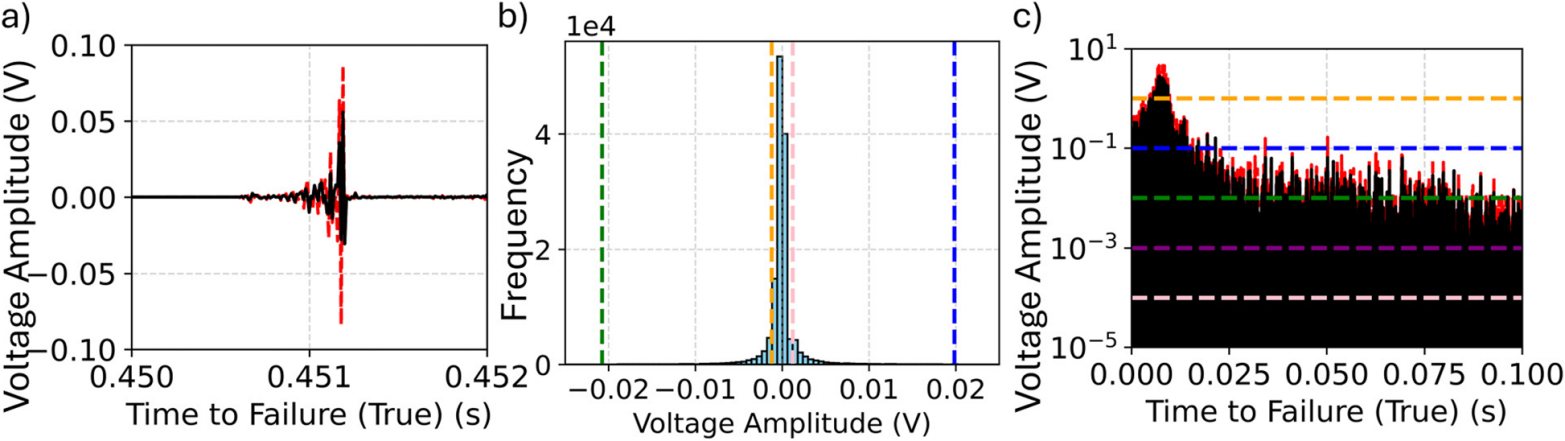
Illustration of extracted features. **a** First Finite Difference of Voltage Amplitude (red) and Voltage Amplitude (black) **b** Histogram of Voltages (light blue) with 1 st (green), 9 th (orange), 91 st (pink), and 99 th (dark blue) Percentiles. **c** Thresholds (yellow – 1, blue – 0.1, green −0.01, purple – 0.001, pink – 0.0001) of First Finite Difference (red) and Voltage (black)

The inputs of the random forest model consist of 64 statistical features calculated from AE signals using a 10 s rolling window. The rolling window unifies the shape of the input samples and is the basis of the engineered statistical features from which the model forecasts the failure time. The calculations are performed for each sensor in each experiment. For each rolling window, statistical features are calculated on the voltage of the AE signal and the first finite difference of the voltage of the AE signals (see Fig. 3a)**.** Features include mean, variance, kurtosis, and skewness and max–min normalized versions of these features; additionally, parametric features include the percentiles (1%−9%, 91%−99%) (see Fig. 3b), time over threshold (1, 10^–1^, 10^–2^, 10^–3^, and 10^–4^ V) (see Fig. 3c), minimum value, and maximum value.

Data imputation is done by removing rows containing non-numeric values and replacing infinite values with the maximum finite value of 1 × 10^99^, since the random forest model can only consider finite values. Next, we identify outlier rows by calculating the Modified Z-Scores [23] for each statistical calculation, and we remove rows corresponding to Modified Z-Scores greater than or equal to 10. We note the usage of z-scores implicitly assumes the voltage to be Gaussian. This is generally true for energy-related AE parameters, but we acknowledge that some AE parameters may be better fit with other probability distributions, such as the Weibull distribution [24]. We transform the time to failure in each experiment into logarithmic space to place heavier weighting on time differences close to failure and lighter weighting on time differences farther from failure. Finally, we normalize the input and output using a standard scaler to improve the numerical stability of the machine learning model [23].

Of note, there are four primary differences between the calculations performed here and those done by Rouet-Leduc et al. [9]: 1) they used Fourier calculations and autocorrelation features and suggested these input features have low importance, so Fourier calculations and autocorrelations features are not used here. 2) Their experiments investigated time to failure forecasting of multiple stick slip cycles, whereas our work focuses on the time to failure forecasting of a single specimen-scale fracture induced by fluid injection. Mechanistically, they are interested in type II (in-plane shear) fractures whereas we are interested in type I (tensile opening) fractures. 3) We choose the threshold values (1, 10^–1^, 10^–2^, 10^–3^, and 10^–4^ V) based on the amplitude of our AE signal, which are different from theirs (10^–9^, 5 × 10^–9^, 10^–8^, 5 × 10^–8^, and 10^–7^ V). 4) They used a rolling window of 1.8 s with intervals of 0.18 s whereas we use a rolling window of 10 s with intervals of 0.1 s as our experiments occur over a longer duration and at a higher sampling rate. We also tested 0.5 and 1.8 s windows but found the best performance with 10 s windows.

#### Time to failure

**Fig. 4:**
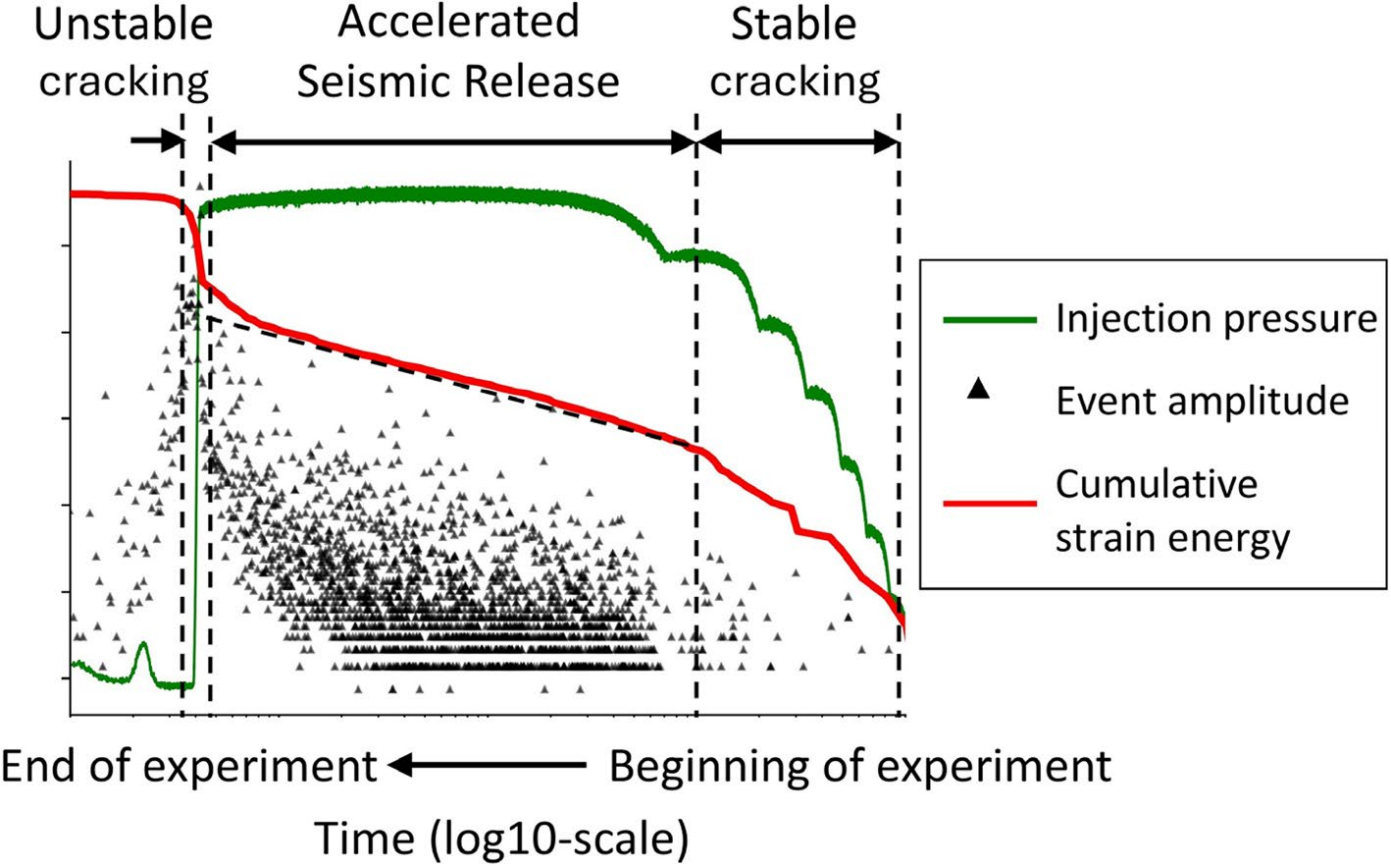
Schematic plot of AE events and injection pressure evolution during the fluid injection

The model output is the time to failure. Here, failure refers to the initiation of unstable fracturing as outlined in Fig. 4, where we see a sudden drop in injection pressure accompanied by a rapid increase in released cumulative strain energy (red curve). The accelerated seismic release stage precedes unstable fracturing, where microfractures initiate and propagate at an increasing speed following a linear tendency with log-time [21, 25]. The injection pressure can be viewed analogously to the axial load in a classic uniaxial compression test.

**Fig. 5:**
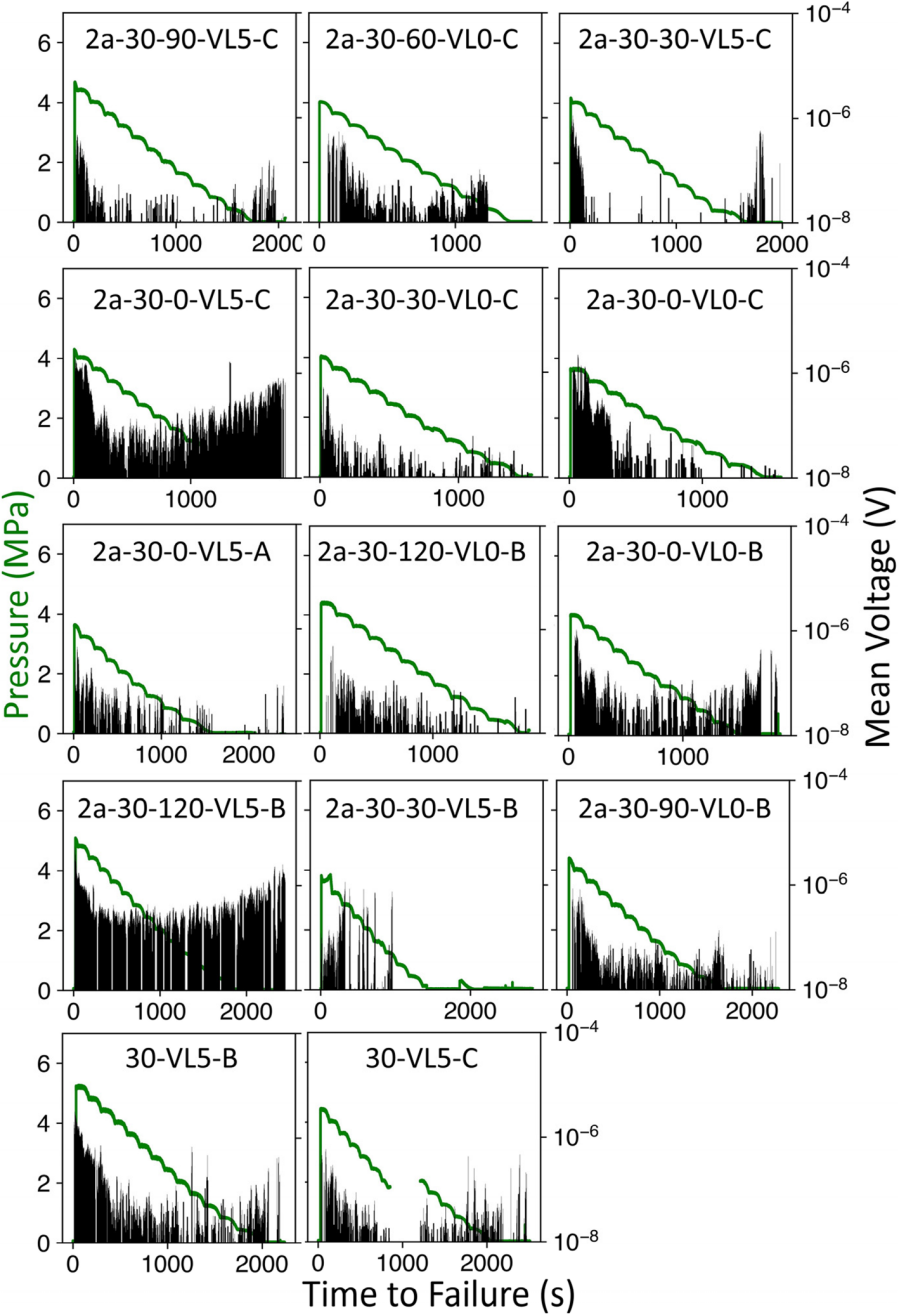
Injection pressure (green) and Mean Voltage (black) over Time to Failure for individual experiments after 10σ outlier removal. The voltages corresponding to the period of missing pressure data in Experiment 30-VL5-C are excluded from model training and testing

Figure 5 illustrates the mean voltages over the time to failure for individual experiments used in model training and testing together with injection pressure as a reference. The events at early times occur due to the initial vertical loading. We eliminate outlier data beyond 10σ from the mean value of each experiment. Note that some of the pressure data were not recorded during experiment 30-VL5-C between 853.1 to 1210.8 s prior to failure. This occurred during the stable fracturing stage (see Fig. 4) and has a negligible effect on initiation of unstable fracturing. Additionally, our model only uses voltage related features as input. Thus, we exclude the voltage data when pressures are unavailable. All experiments are loaded to failure through water pressure. We note that the dataset is diverse in that it includes numerous flaws geometries that induce dramatically different stress regimes [26]. This diversity incentivizes our model to learn more generalized correlations between the AE waveforms and TTF.

#### Model and evaluation

We implement the random forest model using the python Scikit-Learn package as the regression tool to forecast the rock specimen’s closeness to failure represented by the time to failure. The inputs are the AE signals represented by summary statistical features. A random forest [27] consists of a collection of decision trees that use a series of tests (represented by internal nodes) to split the dataset based on the test outcome (represented by branches), where the Gini index is used to assess the quality of each split [23]. The model then accumulates the results from each decision tree and the final output is the weighted mean of the decision trees in the random forest. The model uses mean squared error as the loss function between the forecasted and true time to failure [23]. The random forest models are optimized using a five-fold cross-validation grid search on combinations of 1) the number of trees as 50, 100, 150, and 2) maximal tree depth as 5, 10, and unlimited. The data are split into 50% Training/50% Testing to confirm the robustness of the trained mode. To evaluate the performance of the model, we use the coefficient of determination *R*^2^ as a Goodness of Fit indicator and plot the forecasted time to failure against the true time to failure.

## Results and discussion

### Model performance

**Fig. 6:**
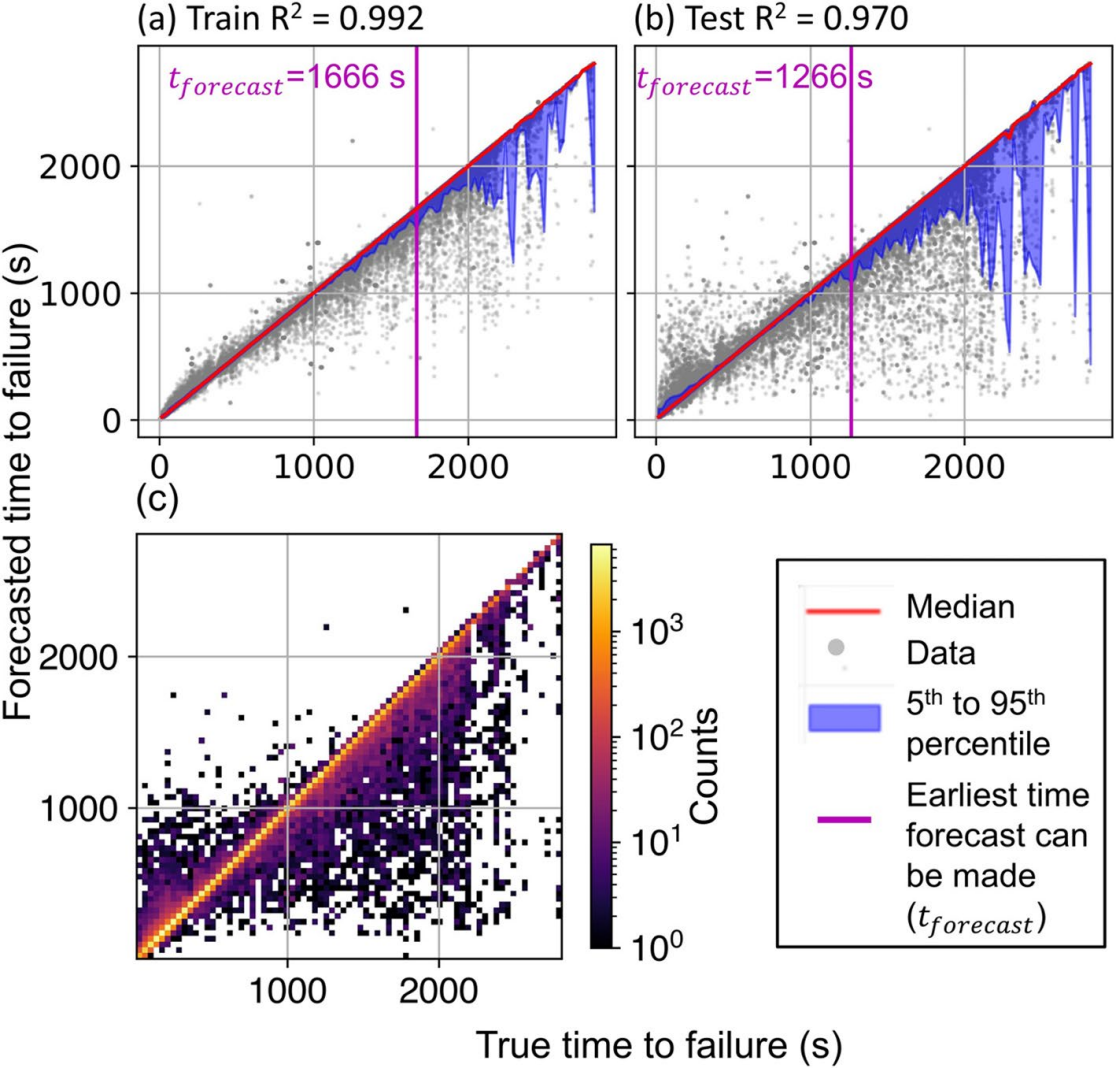
Time to Failure (Forecasted) vs. Time to Failure (True) on (**a**) training and (**b**) testing dataset. The blue band indicates the 5 th to 95 th percentile of the data in 25 s bins. Magenta line and text indicate earliest time *t*_forecast_ when the 90-percentile prediction exceeds 75 s. (**c**) Density plot of panel (**b**) using 75-s bins

Figure 6 shows that both training and testing data exhibit a near 1:1 relationship between median forecasted and true time to failure. The blue band illustrates the 90 th percentile band (5 th to 95 th percentile) of forecasted TTF within each 25-s bin. We define ${t}_{\text{forecast}}$ as the earliest TTF when the forecasted TTF bound exceeds 75 s. The model accurately forecasts TTF until 1666 s prior to failure on the training data and 1266 s prior to failure on the testing data. Additionally, the model predominantly underestimates TTF, forecasting failure on average 9 s earlier than the actual time.

The true-versus-forecasted TTF samples predominantly distribute along the 1:1 diagonal line as shown in Fig. 6(c). This is also true for individual experiments. We adopt the scatter plot to present subsequent results, since they focus on the uncertainty illustrated by the percentile band.

**Fig. 7:**
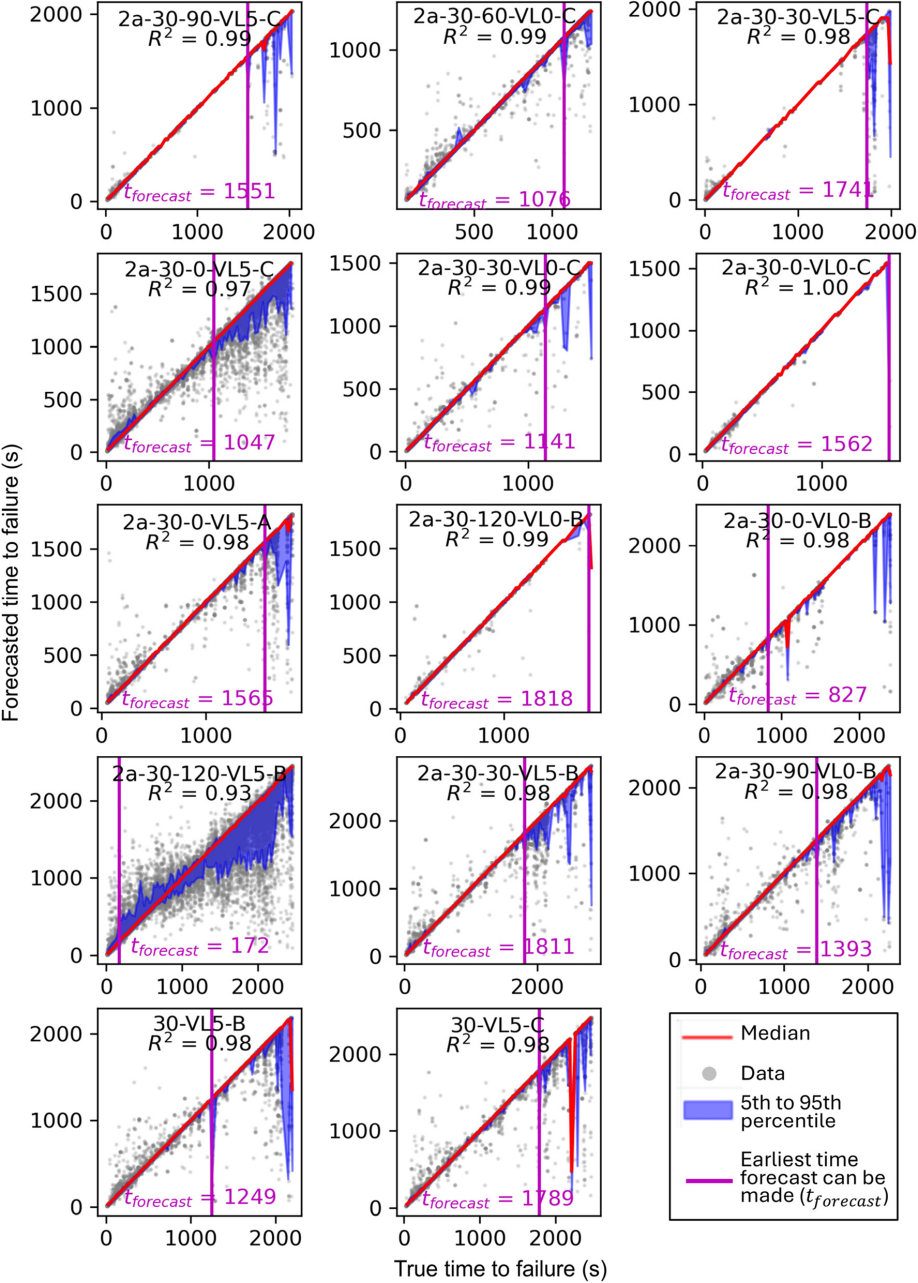
Forecasted vs. True Time to Failure

Model performance on individual experiments is shown in Fig. 7. Generally, all experiments show satisfying *R*^2^ ranging from 0.93 to 0.99. Experiment 2a-30–120-VL5-B has the lowest *R*^2^ as well as the smallest ${t}_{\text{forecast}}$. This is potentially due to low signal to noise ratio caused by inappropriate amplification or sensor coupling. As illustrated in Fig. 5, the noise floor of the mean voltage recorded during experiment 2a-30–120-VL5-B is on the order of 10^–7^ V compared to the majority of other experiments exhibiting a noise floor on the order of 10^–8^ V.

### Input feature importance

Random forest models intrinsically provide a measure of feature importance that can be used for a physical interpretation of the model. Table 1 demonstrates ranked feature importance, which shows that the mean voltage is the best forecast indicator of time to failure. However, Fig. 5 demonstrates the relationship between injection pressure and mean voltage, which shows that mean voltage exhibits periods of high activity that do not correspond to impending failure despite generally increasing towards failure. Thus, the mean voltage by itself is not sufficient to reasonably forecast the time to failure and additional constraints are needed from the other statistical features; specifically, the second most important feature is the minimum amplitude of the first finite difference, which indicates that large changes in voltage occur upon approaching specimen-scale rupture of the rock. This is supported by the ablation tests, where we remove each given feature to quantify the resulting loss in performance. The results presented in Table 1 show that the test *R*^2^ is not strongly dependent on any given feature, for example the test *R*^2^ only decreases from 97% to 96.49% with the removal of the mean voltage. A further observation is that the 20 features shown in Table 1 account for 100% of the total feature importance, and so the remaining 44 features can be disregarded in terms of impact on forecasting the time to failure for the given experimental setup.

**Table 1:** Features with non-zero importance and results of ablation tests showing the *R*^2^ on the testing and training data if the feature is removed

Feature	Feature Importance	Test *R*^2^	Train *R*^2^
Mean	0.779	96.49%	99.55%
Min Strain Amplitude of First Finite Difference	0.218	96.85%	99.59%
Kurtosis of First Finite Difference	0.202	96.75%	99.59%
Kurtosis of First Finite Difference (Not Normalized)	0.188	96.75%	99.59%
Skewness (Not Normalized)	0.171	96.74%	99.59%
Skewness	0.166	96.74%	99.59%
Kurtosis	0.137	96.75%	99.59%
Kurtosis (Not Normalized)	0.135	96.75%	99.59%
Variance	0.134	96.76%	99.59%
Variance (Not Normalized)	0.127	96.76%	99.59%
Threshold 0.0001 of First Finite Difference	0.126	96.95%	99.60%
Skewness of First Finite Difference (Not Normalized)	0.114	96.75%	99.59%
Max Strain Amplitude of First Finite Difference	0.114	96.87%	99.59%
Skewness of First Finite Difference	0.109	96.75%	99.59%
Threshold 0.0001	0.105	96.75%	99.59%
Min Strain Amplitude	0.030	96.89%	99.60%
Max Strain Amplitude	0.027	96.93%	99.60%
Variance of First Finite Difference (Not Normalized)	0.010	96.77%	99.59%
Variance of First Finite Difference	0.010	96.77%	99.59%
Mean of First Finite Difference	0.000	96.75%	99.59%

**Fig. 8:**
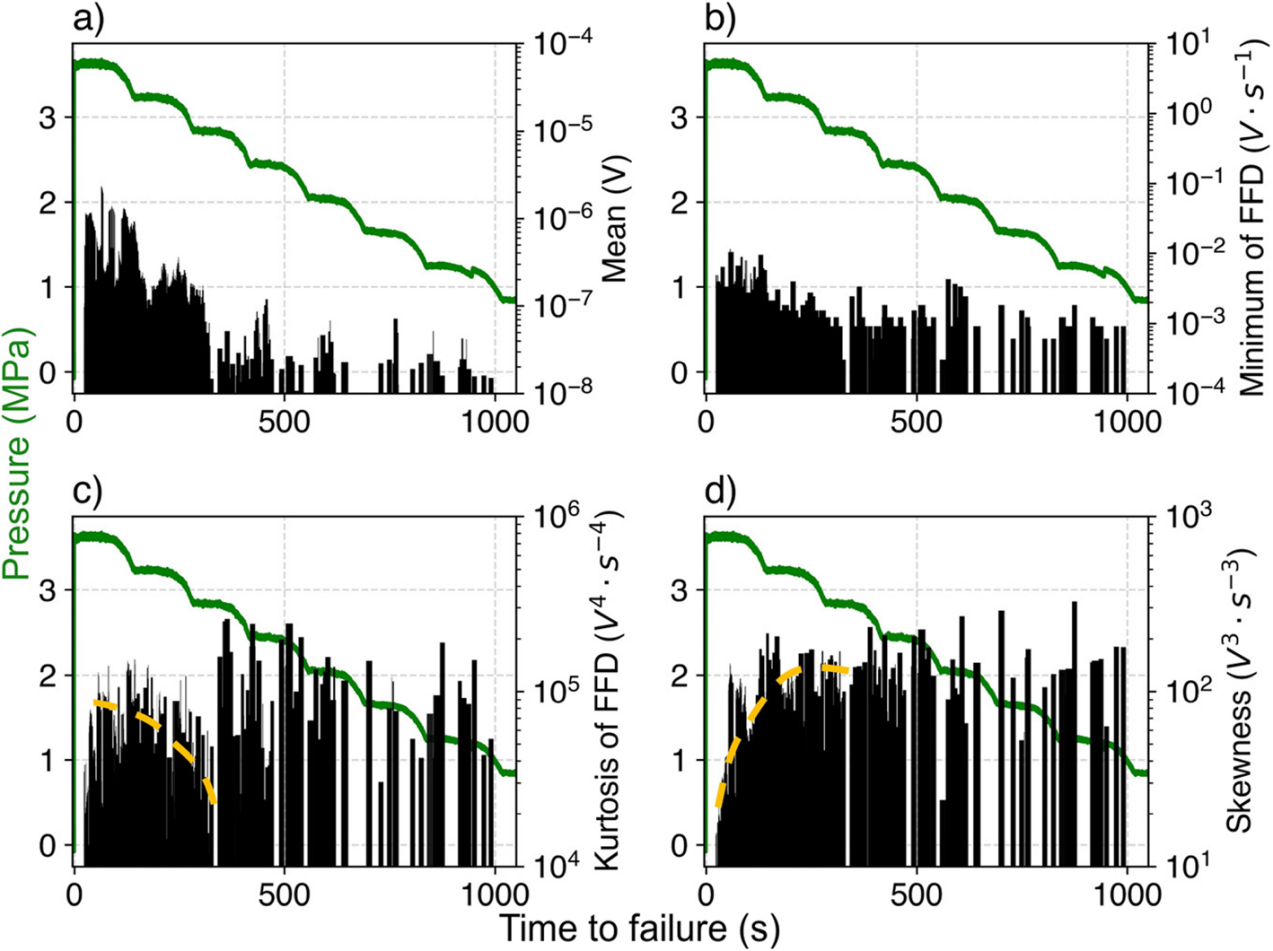
Four key statistical features of experiments experiment 2a-30–0-VL0-C versus injection pressure. (**a**) Mean amplitude (**b**) Minimum of first finite difference (FFD) (c) Kurtosis of FFD (d) Skewness. The yellow dashed lines in (**c**) and (**d**) indicate transition from unrelated microfractures to unstable fracturing

Figure 8 demonstrates how these high-ranking features behave throughout an experiment, showing that all features contribute significantly to the forecast of the time to failure. It is notable that all four statistical moments (mean, variance, skewness, kurtosis) rank highly. The mean amplitude (Fig. 8a) generally increases monotonically towards failure, indicating the increasing average energy of the system as the water pressure increases. The first finite difference (FFD) reflects the difference between consecutive measured amplitudes. The minimum of FFD (Fig. 8b) implies a baseline entropy of the system which also appears to increase when the rock reaches a state of accelerated seismic release. Meanwhile, Kurtosis of FFD characterizes the level of dispersion, exhibiting a stepwise drop ~ 300 s prior to failure (yellow dashed line in Fig. 8c) while gradually increasing towards failure owing to the rise in entropy caused by bursts of unrelated microfractures, but gradually converges to a dominant proportion of large amplitude events. The decrease in skewness of amplitude prior to 300 s (yellow dashed line in Fig. 8d) also illustrates the increasing proportion of high amplitude events, i.e., macrofractures, after the burst of AE events, which is in agreement with studies showing the AE b-value decreases as the system approaches failure. These provide a qualitative interpretation of the quantitative but physically interpretable forecasting model.

### Comparison to other methods

#### Inverse AE rate

**Fig. 9:**
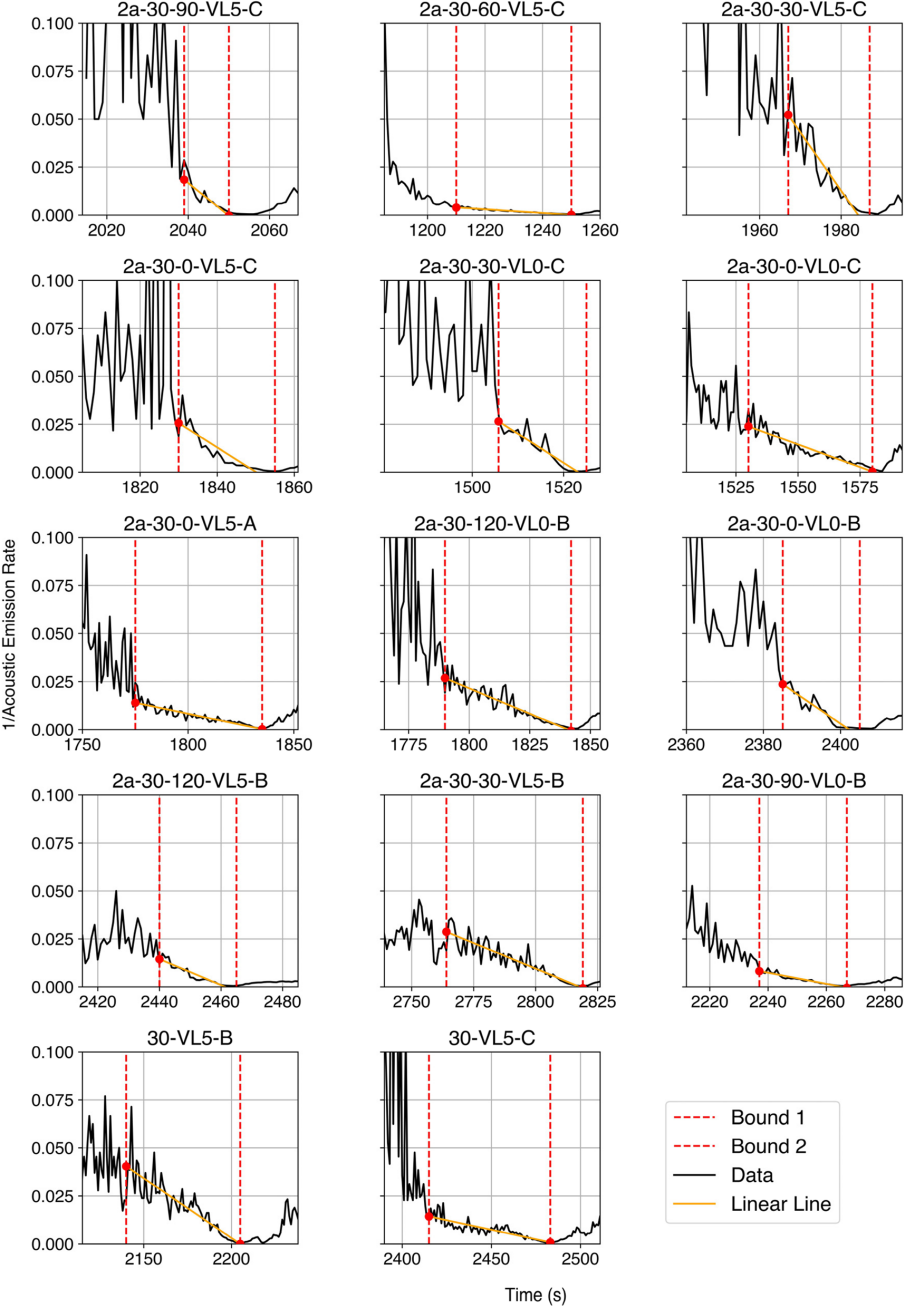
Failure time found through Inverse AE Rate

Researchers have demonstrated inverse velocity as a method to forecast geotechnical events, specifically the time of rock slope instability [28–30]. It has been observed that the inverse of the velocity of the slope, 1/v, exhibits a linear trend on approaching failure (yellow lines in Fig. 9), where the linear trend’s intersection with the abscissa, i.e. when the slope velocity approaches infinity, is its approximate failure time. To relate this to AE, Kim et al. [31] showed that, at stress levels above the crack initiation stress, the cumulative AE energy and cumulative AE moment approximate the cumulative volumetric strain in rocks.

Here, the inverse velocity method is applied to the fourteen hydraulic fracturing experiments using the Acoustic Emission (AE) hit rate as an analogue to velocity [32]. This serves as a benchmark to the machine learning model. The AE hit rate combined across all 8 channels is binned on 1 s intervals, and the reciprocal of this 1-s AE rate is taken as the 1/(AE Rate), as shown in Fig. 9.

The inverse AE rate is initially noisy without a clear trend but converge onto a linear or concave trend on the order of 40 s prior to the failure event. This convergence of the inverse AE rate may indicate a transition from the initiation and propagation of unconnected microcracks to their coalescence into a larger specimen-scale crack that propagates unstably towards the edges of the specimen [33]. This transition from stable microfracturing to accelerated seismic release has been observed in a range of brittle materials [34, 35]. This accelerated seismic release is marked by the rate of AE events reaching a critical state where it transitions from a random set of nucleation times to a power-law evolution controlled by the degree of heterogeneity reflecting the availability of low-strength microcracks.

Table 2 presents the performance of the random forest and inverse AE rate models by the earliest time at which a forecast can be made (*t*_forecast_) and the mean error between the forecasted and true time to failure. For inverse AE rate, *t*_forecast_ is defined as the time when the 1/(AE rate) converges onto a clear trend shown in the left red bounds in Fig. 9.

**Table 2:** The earliest time which models can accurately forecast unstable fracture propagation (*t*_forecast_) and the corresponding mean error of individual experiments. Percentage indicates the ratio of *t*_forecast_ to total experiment duration

Experiment name	Model			
	Random forest		Inverse AE rate	
	*t*_forecast _(s)	Error (s)	*t*_forecast_ (s)	Error (s)
2a-30–90-VL5-C	1550.9 (75.41%)	4.9	18	7
2a-30–60-VL5-C	1075.7 (85.85%)	2.1	43	1
2a-30–30-VL5-C	1740.8 (87.49%)	13.9	23	6
2a-30–0-VL5-C	1047.3 (56.43%)	13.9	26	6
2a-30–30-VL0-C	1141.2 (74.84%)	2.3	19	2
2a-30–0-VL0-C	1561.9 (98.58%)	0.6	54	3
2a-30–0-VL5-A	1564.8 (85.21%)	6.2	61	0
2a-30–120-VL0-B	1818 (98.53%)	0.2	55	4
2a-30–0-VL0-B	827.5 (34.35%)	5.2	24	7
2a-30–120-VL5-B	171.6 (6.96%)	37.8	25	4
2a-30–30-VL5-B	1811.2 (64.24%)	10.7	55	2
2a-30–90-VL0-B	1393.4 (61.46%)	5.3	30	2
30-VL5-B	1248.9 (56.61%)	6.1	66	1
30-VL5-C	1789.3 (72.04%)	8.6	69	3

The results show that random forest models significantly outperform the inverse AE rate method in terms of *t*_forecast_, with the caveat that the error is increased over this larger window. This can be attributed to the subtle information extracted from the raw AE signal. The raw signal, which is readily available in large quantities, provides key insights into the stress state at the scale of individual mineral grains, while the inverse AE rate method only uses the specimen-scale information summarized by the average rate of events. Thus, our results suggest that it is possible to forecast failure of a rock specimen prior to the onset of accelerated seismic release, with implications for managing induced seismicity hazards. However, it is noted that the threshold of *t*_forecast_ for the random forest model is arbitrarily defined to be 75 s and the generalization of this requires further investigation. For example, the trained model presented here would likely not be applicable to other types of loading regimes such as direct shear, (in)direct tension, or uni/triaxial setups; other rock types such as sandstone, limestone, or schist; or larger length-scales where the amplitude and statistical features of the signal would be different from those included in the training dataset.

#### Support vector machine

**Fig. 10:**
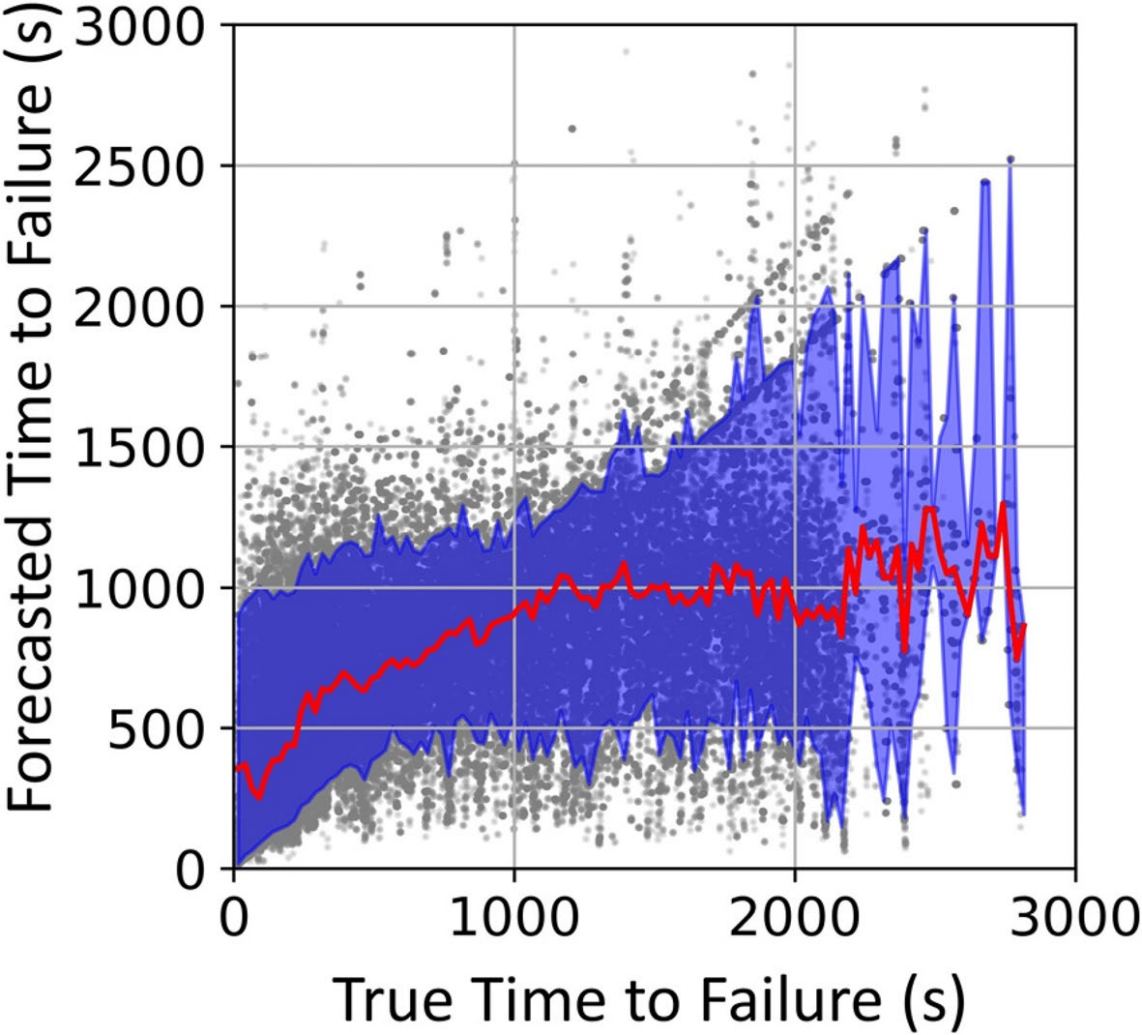
Forecasted vs. True Time to Failure on test set using a support vector machine. Legend follows Fig. 7

We also compare our random forest model to a support vector machine model using the same training and test data. A radial basis function is used as the kernel, and a five-fold cross-validation search found that *C* = 1 and *γ* = 0.1 returns the lowest mean squared error. However, this model results in highly inaccurate forecasts with a coefficient of determination of *R*^2^ of only 0.22. The majority of forecasted TTF are lower than true TTF with ~ 1000 s between the 5 th and 95 th percentile bound as shown in Fig. 10. This suggests that the nature of time to failure forecasting of hydraulic fracturing in rocks is highly non-linear. Specifically, we posit that the poor performance of the support vector machine can be attributed to the fundamental assumption that the regression can be formulated as a single continuous hyper-plane in high dimensional space to make forecasting via some measure of “distance” between datapoints. On the other hand, the numerous decision trees forming a random forest can model highly non-linear behavior owing to its formulation of the problem into discrete classifications that are averaged across the trees.

## Conclusion

This research demonstrates that machine learning can be used to temporally forecast specimen-scale rock fractures caused by hydraulic fracturing. Specifically, our random forest model achieves a coefficient of determination *R*^2^ of 0.97 on test data using a 50% Training/50% Testing split. An analysis of the feature importance of this physically interpretable model demonstrates that 20 statistical features constitute 100% of the contribution to the forecast, where the top 4 features are mean, minimum, kurtosis of the first finite difference, and skewness of the signal voltage, accounting for 48% of total feature importance. The model is generally robust against the importance of individual features, where the removal of the mean as an input only reduces the *R*^2^ on test data from 0.97 to 0.965. Evidently, nuances in energy state play an important role in time to failure forecasting. Our model achieves similar accuracy (generally less than 10 s forecast error) when benchmarked against the inverse AE rate method but presents a significant advantage that it can make accurate forecasting up to 1800 s before the failure event compared to 41 s ahead of the failure event from the inverse AE rate method. We also find poor forecasting ability when using a support vector machine in place of the random forest, suggesting that the forecasting of time to failure of hydraulic fractures is a highly discontinuous problem.

## Data Availability

The data, models, and code required to reproduce this work are available at Mendeley Data (10.17632/8scy63d3tw) [36]. The data include acoustic emission seismic waveforms associated with time to failure parsed from Li et al. [22]'s hydraulic fracturing experiments and statistical features extracted following Rouet-Leduc et al. [9]. The code to load the data and trained models is enclosed in the Jupyter Notebook.

## References

[1] Bolinger M, Millstein D, Gorman W, Dobson P, Jeong S (2023) Mind the gap: Comparing the net value of geothermal, wind, solar, and solar+storage in the Western United States. Renewable Energy 205:999–1009. 10.1016/j.renene.2023.02.023

[2] Jenkins JD, Luke M, Thernstrom S (2018) Getting to zero carbon emissions in the electric power sector. Joule 2(12):2498–2510. 10.1016/j.joule.2018.11.013

[3] Kim KH, Ree JH, Kim Y, Kim S, Kang SY, Seo W (2018) Assessing whether the 2017 Mw 5.4 Pohang earthquake in South Korea was an induced event. Science 360(6392):1007–1009. 10.1126/science.aat6081 29700224 10.1126/science.aat6081

[4] Naik SP, Gwon O, Porfido S, Park K, Jin K, Kim YS, Kyung JB (2020) Intensity reassessment of the 2017 Pohang Earthquake Mw = 5.4 (South Korea) Using ESI-07 scale. Geosciences 10(11):471. 10.3390/geosciences10110471

[5] Jackson DD, Kagan YY (2006) The 2004 parkfield earthquake, the 1985 prediction, and characteristic earthquakes: lessons for the future. Bull Seismol Soc Am 96(4B):S397–S409. 10.1785/0120050821

[6] Bakun WH, Lindh AG (1985) The Parkfield, California, earthquake prediction experiment. Science 229(4714):619–624. 10.1126/science.229.4714.619 17739363 10.1126/science.229.4714.619

[7] Rouet-Leduc B, Hulbert C, Bolton DC, Ren CX, Riviere J, Marone C et al (2018) Estimating fault friction from seismic signals in the laboratory. Geophys Res Lett 45(3):1321–1329. 10.1002/2017GL076708

[8] Mousavi SM, Beroza GC (2023) Machine learning in earthquake seismology. Annu Rev Earth Planet Sci 51(1):105–129. 10.1146/annurev-earth-071822-100323

[9] Rouet-Leduc B, Hulbert C, Lubbers N, Barros K, Humphreys CJ, Johnson PA (2017) Machine learning predicts laboratory earthquakes. Geophys Res Lett 44(18):9276–9282. 10.1002/2017GL074677

[10] Laurenti L, Tinti E, Galasso F, Franco L, Marone C (2022) Deep learning for laboratory earthquake prediction and autoregressive forecasting of fault zone stress. Earth Planet Sci Lett 598:117825. 10.1016/j.epsl.2022.117825

[11] Wang K, Johnson CW, Bennett KC, Johnson PA (2021) Predicting fault slip via transfer learning. Nat Commun 12(1):7319. 10.1038/s41467-021-27553-5 34916491 10.1038/s41467-021-27553-5 PMC8677738

[12] Reches Z, Fineberg J (2023) Earthquakes as dynamic fracture phenomena. J Geophys Res 128(3):e2022JB026295. 10.1029/2022JB026295

[13] Bao X, Eaton DW (2016) Fault activation by hydraulic fracturing in western Canada. Science 354(6318):1406–1409. 10.1126/science.aag2583 27856850 10.1126/science.aag2583

[14] Zeng Y, Cheng W, Zhang X, Xiao B (2020) A criterion for identifying a mixed-mode I/II hydraulic fracture crossing a natural fracture in the subsurface. Energy Explor Exploit 38(6):2507–2520. 10.1177/0144598720923781

[15] Schultz R, Skoumal RJ, Brudzinski MR, Eaton D, Baptie B, Ellsworth W (2020) Hydraulic fracturing-induced seismicity. Rev Geophys 58(3):e2019RG000695. 10.1029/2019RG000695

[16] He Q, Suorineni FT, Oh J (2016) Review of hydraulic fracturing for preconditioning in cave mining. Rock Mech Rock Eng 49(12):4893–4910. 10.1007/s00603-016-1075-0

[17] Kang H, Lv H, Gao F, Meng X, Feng Y (2018) Understanding mechanisms of destressing mining-induced stresses using hydraulic fracturing. Int J Coal Geol 196:19–28. 10.1016/j.coal.2018.06.023

[18] Kang H, Jiang P, Feng Y, Gao F, Zhang Z, Liu X (2023) Application of large-scale hydraulic fracturing for reducing mining-induced stress and microseismic events: a comprehensive case study. Rock Mech Rock Eng 56(2):1399–1413. 10.1007/s00603-022-03061-w

[19] Wang Q, Chen X, Jha AN, Rogers H (2014) Natural gas from shale formation – the evolution, evidences and challenges of shale gas revolution in United States. Renew Sustain Energy Rev 30:1–28. 10.1016/j.rser.2013.08.065

[20] Hwang B, Heo J, Lim C, Park J (2023) Environmental implications of shale gas hydraulic fracturing: a comprehensive review on water contamination and seismic activity in the United States. Water 15(19):3334

[21] Broberg KB (1973) Discussion of initial and subsequent crack growth. Eng Fract Mech 5(4):1031–1035. 10.1016/0013-7944(73)90071-4

[22] Li BQ, Gonçalves da Silva B, Einstein H (2019) Laboratory hydraulic fracturing of granite: acoustic emission observations and interpretation. Eng Fract Mech 209:200–220. 10.1016/j.engfracmech.2019.01.034

[23] Pedregosa F, Varoquaux G, Gramfort A, Michel V, Thirion B, Grisel O et al (2011) Scikit-learn: machine learning in python. J Mach Learn Res 12(85):2825–2830

[24] Sagar RV, Deepak S, Desai PR et al (2019) Statistical analysis of acoustic emissions generated during unconfined uniaxial compression of cementitious materials. Constr Build Mater 225:692–708

[25] Li BQ, Einstein HH (2019) Direct and microseismic observations of hydraulic fracturing in Barre Granite and Opalinus Clayshale. J Geophys Res 124(11):11900–11916. 10.1029/2019JB018376

[26] Gonçalves da Silva B, Einstein HH (2014) Finite Element study of fracture initiation in flaws subject to internal fluid pressure and vertical stress. Int J Solids Struct 51(23):4122–4136. 10.1016/j.ijsolstr.2014.08.006

[27] Breiman L (2001) Random forests. Mach Learn 45:5–32

[28] Carlà T, Intrieri E, Di Traglia F, Nolesini T, Gigli G, Casagli N (2017) Guidelines on the use of inverse velocity method as a tool for setting alarm thresholds and forecasting landslides and structure collapses. Landslides 14(2):517–534. 10.1007/s10346-016-0731-5

[29] Fukuzono T (1985) A method to predict the time of slope failure caused by rainfall using the inverse number of velocity of surface displacement. Landslides 22(2):8-13_1. 10.3313/jls1964.22.2_8

[30] Zhang J, Yao H, Wang Z, Xue Y, Zhang L (2023) On prediction of slope failure time with the inverse velocity method. Georisk: Assessment and Management of Risk for Engineered Systems and Geohazards. Retrieved from https://www.tandfonline.com/doi/full/10.1080/17499518.2022.2132263

[31] Kim JS, Lee KS, Cho WJ, Choi HJ, Cho GC (2015) A Comparative evaluation of stress-strain and acoustic emission methods for quantitative damage assessments of brittle rock. Rock Mech Rock Eng 48(2):495–508. 10.1007/s00603-014-0590-0

[32] Rose ND, Hungr O (2007) Forecasting potential rock slope failure in open pit mines using the inverse-velocity method. Int J Rock Mech Min Sci 44(2):308–320. 10.1016/j.ijrmms.2006.07.014

[33] Gonçalves da Silva B, Einstein H (2018) Physical processes involved in the laboratory hydraulic fracturing of granite: Visual observations and interpretation. Eng Fract Mech 191:125–142. 10.1016/j.engfracmech.2018.01.011

[34] Patton A, Goebel T, Kwiatek G, Davidsen J (2023) Large-scale heterogeneities can alter the characteristics of compressive failure and accelerated seismic release. Phys Rev E 108(1):014131. 10.1103/PhysRevE.108.014131 37583189 10.1103/PhysRevE.108.014131

[35] Vu CC (2019) Compressive failure as a critical transition: experimental evidence and mapping onto the universality class of Depinning. Physical Review Letters 122(1):015502. 10.1103/PhysRevLett.122.015502 31012687 10.1103/PhysRevLett.122.015502

[36] Hooper M, Xie AY, Li Q (2024) random forest prediction of time to failure for granite hydraulic fracturing using nanoseismic signals [Data set]. Mendeley Data 9:100129. 10.17632/8scy63d3tw.2

